# Regional Anatomy in Its Relation to Medicine and Surgery

**Published:** 1892-09

**Authors:** 


Regional Anatomy in Its Relation to Medicine and Surgery. By George McClellan, M. D., Lecturer on Descriptive and Regional Anatomy at the Pennsylvania School of Anatomy; Professor of Anatomy at the Pennsylvania Academy of the Fine Arts; Member of the Association of American Anatomists, Academy of Natural Sciences, Academy of Surgery, College of Physicians, etc., Philadelphia. Illustrated from photographs taken by the author of his own dissections, expressly designed and prepared for this work, and colored by him after Nature. In two volumes. Volume II., quarto, pp. xiv.—414. Philadelphia: J. B. Lippincott Co. 1892.

Those already familiar with Vol. I. of this most excellent treatise have been expectantly awaiting the appearance of Vol. II. Doubtless much curiosity has existed as to whether the second volume would equal in merit its predecessor. Those who had formed a high estimate of the latter will suffer no disappointment upon perusal of the volume now at hand. It sustains fully the reputation which Vol. I. gained for the author.

The opening pages take up the abdomen and its contents, and are replete with good practical anatomy. Especially commendable from this point of view is the treatment of the surface anatomy of the abdominal walls, the information given being a judicious combination of existing literature and the personal experience of a surgeon and anatomist. The author then discusses, in the same practical way, the inguinal, pelvic, and perineal regions. These regions afford him an excellent opportunity for displaying his powers as a writer of regional anatomy, as we commonly understand this term, and we are sure the practitioner will find a mine of useful and correct information in the pages devoted to these portions of the human frame. The back, lumbar and gluteal regions, the hip and lower extremity complete the volume, and here, as elsewhere, the pages fairly bristle with applications of the anatomy given to practical medicine and surgery. The operations common to these various regions such as those upon the kidney and colon, the different amputations, fractures and dislocations, and other surgical conditions occurring in these parts, are given in detail. One forgets at times that he is reading an anatomy at all and could readily imagine his pages belonging to a work on surgery.

There are two states of mind which can lead the reviewer of a first-class book like this to temper his praise. The one, a very common one, is that no review is complete unless some fault has been found, and incidentally the reviewer takes occasion to air his ability as a critic. The other has its origin in an inherent desire in the human mind to have that which is so nearly perfect entirely so, and a criticism actuated by such a motive is meant solely for the good of the author and his book. It is the latter state of mind which allows us to venture the opinion that in this most admirable work the author has now and then let slip an opportunity to keep the pages of his book of equal merit. A case or two in point: Upon page 222, in describing the *gluteus maximus* muscle, the following statement occurs: "This muscle is supplied by the lesser sciatic nerve, etc." The author makes no comment upon the fact that this nerve is essentially concerned in furnishing sensation to the integument over the genitalia, and that, in supplying a muscle whose companions, the *medius* and *minimus*, get their nerve supply from a different source (the superior gluteal), there must be some good and sufficient reason, which, according to Hilton, is that the action of this muscle in its relation to coitus makes it necessary that it be in sympathy with the genitalia, and, hence, they have a common nerve supply.

Again, in speaking of the ligamentum teres, nothing is said of Tillaux's theory of its function, to our mind the best that has been advanced, viz.: that it acts as a buffer between the head of the femur and the bottom of the acetabulum. Dr. McClellan thinks it may render some slight assistance to muscles steadying the pelvis in the erect position. The point, to be sure, is not a great one, but where little is known upon any subject, it is, in our opinion, worth while to tell all that is reasonable that is known about it.

But deficiencies such as these are only slight blemishes and do not really impair the worth and usefulness of the work, which has been constructed in the main out of the best material and by a most competent architect.

The publishers have left nothing to criticise in the general dress of the book. The plates are quite equal to those of Vol. I., and while we adhere to the opinion expressed in a review of Vol. I., that oftentimes a schematic representation serves a better purpose than photographs of dissections, still the latter have their place and many of these are certainly models of their kind.

